# Canadian Consensus Statements on the Transition of Adolescents and Young Adults with Inflammatory Bowel Disease from Pediatric to Adult Care: A Collaborative Initiative Between the Canadian IBD Transition Network and Crohn’s and Colitis Canada

**DOI:** 10.1093/jcag/gwab050

**Published:** 2022-03-26

**Authors:** Nancy Fu, Natasha Bollegala, Kevan Jacobson, Karen I Kroeker, Karen Frost, Waqqas Afif, Wael El-Matary, Sharyle A Fowler, Anne M Griffiths, Hien Q Huynh, Prévost Jantchou, Ahmer Karimuddin, Geoffrey C Nguyen, Anthony R Otley, Christina Pears, Cynthia H Seow, Alene Toulany, Claudia Tersigni, Joanne Tignanelli, John K Marshall, Monica Boctor, Tawnya Hansen, Chandni Pattni, Andrew Wong, Eric I Benchimol

**Affiliations:** Division of Gastroenterology, Department of Medicine, University of British Columbia, Vancouver, British Columbia, Canada; Division of Gastroenterology, Department of Medicine, Women’s College Hospital, Toronto, Ontario, Canada; Division of Gastroenterology, Hepatology and Nutrition, BC Children’s Hospital, Vancouver, British Columbia, Canada; Division of Gastroenterology, Department of Medicine, University of Alberta, Edmonton, Alberta, Canada; Division of Gastroenterology, Hepatology and Nutrition, The Hospital for Sick Children, Toronto, Ontario, Canada; Division of Gastroenterology, Department of Medicine, McGill University, Montreal, Quebec, Canada; Department of Pediatrics and Child Health, University of Manitoba, Winnipeg, Manitoba, Canada; Department of Medicine, University of Saskatchewan, Saskatoon, Saskatchewan, Canada; Division of Gastroenterology, Hepatology and Nutrition, The Hospital for Sick Children, Toronto, Ontario, Canada; Division of Pediatric GI Nutrition, Department of Pediatrics, University of Alberta, Edmonton, Alberta, Canada; Division of Gastroenterology, Department of Pediatrics, CHU Sainte-Justine, Montréal, Quebec, Canada; Department of Surgery, University of British Columbia, Vancouver, British Columbia, Canada; Department of Medicine, Mount Sinai Hospital Inflammatory Bowel Disease Centre, University of Toronto, Toronto, Ontario, Canada; Division of Pediatric Gastroenterology & Nutrition, IWK Health Centre, Halifax, Nova Scotia, Canada; IBD Centre BC, Vancouver, British Columbia, Canada; Division of Gastroenterology and Hepatology, Departments of Medicine and Community Health Sciences, University of Calgary, Calgary, Alberta, Canada; Division of Adolescent Medicine, Department of Paediatrics, University of Toronto, Toronto, Ontario, Canada; Department of Medicine, University of Toronto, Toronto, Ontario, Canada; Patient Partner, Ontario, Canada; Division of Gastroenterology, Farncombe Family Digestive Health Research Institute, McMaster University, Hamilton, Ontario, Canada; Department of Medicine, University of Toronto, Toronto, Ontario, Canada; Department of Medicine, University of Manitoba, Winnipeg, Manitoba, Canada; Department of Medicine, University of Toronto, Toronto, Ontario, Canada; University of British Columbia, Vancouver, British Columbia, Canada; Division of Gastroenterology, Hepatology and Nutrition, The Hospital for Sick Children, Toronto, Ontario, Canada

**Keywords:** Adolescents, Crohn’s disease, Inflammatory bowel disease, Transition, Ulcerative colitis, Young adults

## Abstract

**Objectives:**

With the increased prevalence of childhood-onset inflammatory bowel disease (IBD), there is a greater need for a planned transition process for adolescents and young adults (AYA). The Canadian IBD Transition Network and Crohn’s and Colitis Canada joined in collaborative efforts to describe a set of care consensus statements to provide a framework for transitioning AYA from pediatric to adult care.

**Methods:**

Consensus statements were drafted after focus group meetings and literature reviews. An expert panel consisting of 20 IBD physicians, nurses, surgeon, adolescent medicine physician, as well as patient and caregiver representatives met, discussed and systematically voted. The consensus was reached when greater than 75% of members voted in agreement. When greater than 75% of members rated strong support, the statement was rendered a strong recommendation, suggesting that a clinician should implement the statement for all or most of their clinical practice.

**Results:**

The Canadian expert panel generated 15 consensus statements (9 strong and 6 weak recommendations). Areas of focus of the statements included: transition program implementation, key stakeholders, areas of potential need and gaps in the research.

**Conclusions:**

These consensus statements provide a framework for the transition process. The quality of evidence for these statements was generally low, highlighting the need for further controlled studies to investigate and better define effective strategies for transition in pediatric to adult IBD care.

## INTRODUCTION

Transition is the process of migrating a patient with chronic disease from pediatric to adult care (1). It is characterized by uninterrupted, coordinated, comprehensive care with attention to the clinical, psychosocial and educational/vocational needs of adolescent and young adult (AYA) patients (2,3). Failed transition and transfer is associated with increased emergency department visit, hospitalization, medication escalation, surgery and overall poor patient adherence and attitude (1,4–7).

In 2018, more than 7000 Canadian children were living with inflammatory bowel disease (IBD), with 600 to 650 patients under the age of 16 years diagnosed every year. It is predicted that by 2030, 13,685 children and adolescents will be living with IBD in Canada (8,9). While the importance of the transition period has been recognized by several major societies, there is a paucity of primary research to guide recommendations (10–14).

Due to heterogeneity in clinical practice (15), in 2019, the Canadian IBD Transition nEtwork (CITE) and Crohn’s and Colitis Canada (CCC) initiated a collaboration to understand Canadian practice in transition and develop consensus statements of best practice for transition. Fifteen consensus statements were developed (Table 1). CITE is a multidisciplinary group of Canadian IBD transition-focused health care providers. The statements were to help standardize care delivery, increase provider awareness regarding existing tools and strategies to measure and optimize outcomes, increase multidisciplinary cooperation and collaboration, and create a platform that can be leveraged for further innovation and future research.

**Table 1.** Consensus statements on the transition of adolescents and young adults with inflammatory bowel disease from pediatric to adult care

Statement 1*	All AYA with pediatric-onset IBD should attend a structured transition program.
Statement 2	A structured transition program should incorporate:
	◦-Delivery of personalized care with a multi-disciplinary approach.
	◦-Collaborative goal setting between patients, guardians, and health care providers.
	◦-Communication strategies that are adaptable to the patient, health care provider and local setting.
	◦-A defined post-transfer adult transition phase.
	◦-Evaluation of the program’s processes and outcomes, and change in response to this evaluation.
Statement 3*	Transition programming should be structured according to the local resources and should reflect input from local key stakeholders.
Statement 4	A pediatric to adult IBD transition of care program should implement developmentally appropriate strategies for AYAs to assess and address health-related knowledge, health-related behaviours, and transition-related skills.
Statement 5	A pediatric to adult IBD transition of care program should address IBD-related adolescent issues with AYAs.
Statement 6*	A pediatric to adult IBD transition of care program should implement strategies for parents/guardians to support and encourage the development of independence in AYAs.
Statement 7*	HCP training programs should integrate training in transition and create opportunities for related knowledge and skill development.
Statement 8*	Patients with pediatric onset IBD undergoing transition of care to adult services should have access to a primary care provider.
Statement 9	A pediatric to adult IBD transition of care program should include a transition coordinator/navigator.
Statement 10*	The timing of care transfer to adult services should be flexible. Strategies should be implemented to optimize communication during the handover process between pediatric and adult IBD health care providers.
Statement 11*	Transfer of care documents should be prepared by the pediatric team. These should include a transfer letter summarizing the individualized transition plan and a concise review of the patient’s medical history. Relevant supporting records should be included.
Statement 12	IBD transition of care networks should be developed and supported to facilitate transition and transfer planning.
Statement 13*	The adult team engaged in a structured pediatric to adult IBD transition program should prioritize care delivery to transitioning AYAs.
Statement 14*	The pediatric and adult IBD transition teams should review the processes and structure of adult health care with AYAs and parents/guardians. The adult IBD transition team should establish expectations and goals with the AYAs and parents/guardians.
Statement 15	A pediatric to adult IBD transition of care program should accommodate groups with special needs, with the support of other specialists.

AYA, adolescents and young adults; IBD, inflammatory bowel disease.

*Denotes strong recommendation.

## METHODS

### Scope and Purpose

A series of in-person CITE-led stakeholder meetings were held from 2017 to 2019 involving Canadian adult and pediatric gastroenterologists, nurses, patients, caregivers, as well as stakeholders from CCC. A steering committee, selected by the co-chairs (N.F., N.B.), made up of key stakeholder group members, then selected additional representatives from diverse Canadian geographic regions with a wide breadth of clinical care, research and implementation expertise in transition.

### Literature Search and Appraisal

MEDLINE and EMBASE searches were conducted spanning the literature from inception to June 11, 2019. The search foci were IBD, adolescents, young adults, with literature regarding care transition, transition models, guidelines or position papers reviewed. Other childhood-onset chronic diseases of interest included juvenile rheumatoid arthritis, type 1 diabetes, congenital heart disease, cystic fibrosis, HIV, cerebral palsy, attention-deficit hyperactivity disorder and autism. Search strategies are available in Supplementary Appendix 1. Two authors (N.F., N.B.) conducted an initial screen of available titles and abstracts. Relevant full publications were reviewed. Most studies pertaining to AYA transition were small, non-interventional leading to overall low to very low quality of evidence. Authors (N.F., N.B.) thus elected to conduct a qualitative review process, extracting relevant information on available data as opposed to formal grading. A recently published systemic review on AYA transition in IBD using GRAD noted very low evidence (16). It is used in conjunction with a separate published systematic review on transition models (17).

### Consensus Process

The draft consensus statements were developed based on the results of the literature search and the CITE-CCC stakeholder meetings, by the co-chairs of the steering committee (N.F., N.B.). The full steering committee (K.K., K.J., K.F., E.B.) reviewed and revised these statements in a series of virtual meetings. A final vote was conducted and only statements that reached the majority (>50%) were reviewed by the full expert panel. The statements and relevant literature were circulated to the full voting panel for review and voting before convening the final in-person expert panel meeting.

The expert panel, consisting of 20 IBD-focused individuals representing adult (*n* = 7) and pediatric (*n* = 7) gastroenterology, adult (*n* = 1) and pediatric (*n* = 1) nursing, surgery (*n* = 1) and adolescent medicine (*n* = 1), as well as patient (*n* = 1) and caregiver (*n* = 1) representatives. The panel convened at a face-to-face meeting (Toronto, Canada, November 17, 2019). One voting member did not remain for the entire voting process, and the denominators were adjusted accordingly following his departure. Anonymous voting was conducted using online polling software (Poll Everywhere; www.polleverywhere.com; San Francisco, USA), with each voting member voting as a pediatric gastroenterologist (Ped GI), adult gastroenterologist (Adult GI), or other provider (other).

The statements and supporting literature were each presented to the voting panel followed by discussion. Based on the discussion, the statements were revised as needed before voting. The first vote was to determine whether members agreed with the statement (‘Agreement’) and required 75% or more of voting members to reach a consensus. When a statement did not reach consensus, further discussion followed by either revision and re-vote, or rejection.

A second vote determined the strength of each recommendation (‘Strength’). Votes were cast on whether participants would support the statement (Yes/No). A strong recommendation was adopted with a 75% or greater vote result. The strength of agreement considered the risk and benefit balance for the patient, the clinical experience of the voting member, patient values and preferences, costs, resource availability, practicability, as well as strength of evidence. In most cases, the evidence base was limited to small observational studies. Strong support suggested that a clinician should implement the statement for all or most of their clinical practice unless there was a compelling reason not to do so. Weak support (<75% of the vote) was reserved for activities considered aspirational in nature and goals for future clinical management when resources or other considerations would allow. Two statements pertaining to serial assessments during the transition were removed during the consensus process as they were deemed to be a duplication of current statements. This process was based on the Canadian Gastroenterology Association policy on clinical practice guideline (18).

### Role of Funding Sources

Funding for the preparation of the position statement was provided through a grant from CCC. Representatives from the granting agency attended the consensus meeting but did not vote or participate in the discussion.

## CONSENSUS STATEMENTS

### Transition Program Structure

***Statement 1. All adolescents and young adults (AYA) with pediatric-onset inflammatory bowel disease should attend a structured transition program.*** [Strong recommendation]

Disease knowledge, self-advocacy skills, and autonomy are necessary for AYA patients to effectively navigate the adult health care system. However, AYAs with IBD frequently demonstrate suboptimal ability to recall personal health history, knowledge of medications and the impact of drugs and alcohol on their disease (19–22). They often defer responsibility for scheduling appointments, requesting medication refills or contacting care providers (19–22). They may also have difficulties processing their emotions, managing role changes, coping with living with a chronic illness, and considering their future with IBD (19–22). As a result, patients are at risk for loss-to-follow-up and those diagnosed in late adolescence have higher emergency department use in the post-transfer period (4).

Despite educational efforts to increase pediatric providers’ awareness of issues by AYAs transitioning to adult care, this awareness has not been shown to significantly impact important patient outcomes such as self-reliance (20). However, formal transition programs have been demonstrated to result in more consistent clinic attendance, greater medication adherence, fewer hospital admissions and surgeries after the transfer, and greater achievement in growth potential (5). Patients and caregivers support the participation in formalized transition interventions (22–24). Hait et al. have published suggested transition timelines and milestones (25).

A structured transition program should be implemented for all patients, beginning in early adolescence and extending beyond transfer to adult care. The specific transition program should suit the needs of the patient population and reflect available resources with defined goals that are based on both patients’ chronological and developmental stages. Flexibility should be key in the design as the requisite time needed for adequate skill development may vary amongst the AYAs. For patients diagnosed in late adolescence, an expedited transition process is required. Education and skills development should accommodate the shortened duration of pediatric care and adult providers should be prepared to assess and address health knowledge, transition skills and health-related behaviours. Patients with suspected IBD close to the age at which transfer to adult gastroenterology care would be required should have their initial investigations completed by an adult gastroenterologist, if possible.

***Statement 2. A structured transition program should incorporate:***

- ***Delivery of personalized care with a multi-disciplinary approach.***
- ***Collaborative goal setting between patients, guardians, and health care providers.***
- ***Communication strategies that are adaptable to the patient, health care provider and local setting.***
- ***A defined post-transfer adult transition phase.***
***- An evaluation of the program’s processes and outcomes and change in response to this evaluation.*** [Weak recommendation]

*Delivery of personalized care with a multi-disciplinary approach—*Many transition clinics involving a multidisciplinary team have been reported. An example of this approach was reported at the Tel Aviv Sourasky Medical Center (26,27). The transition process involved three visits with a gastroenterologist and nurse, predefined goals and support from psychology, social work, dietitian, pharmacy and adolescent medicine as required. AYAs achieved high self-efficacy scores in all domains. The average duration of transition was 6.9 ± 3.5 months. Program duration correlated with transition readiness. Another example is, at the British Columbia Children’s Hospital, AYA and family undergone joint transition visits with pediatric and adult gastroenterologists and nurses. In this clinic, the team reviewed medical, therapeutic histories and completed transition plans with AYA and family. All transition-aged patients reported low overall adherence to medical therapy but patients who attended the clinic reported significantly stronger beliefs that medications were necessary (6).

*Collaborative goal setting between patients, guardians, and health care providers—*There can be considerable differences among the perspectives of patients, health care providers and caregivers regarding the definition of and requirements for successful transition (28). Collaborative goal setting has resulted in more positive patient experiences and serves to manage the expectations of key stakeholder groups and focus on salient tasks. Topics of discussion could include the transfer process, future adult health care providers, peer support and mentoring, and setting specific goals and timelines before transfer (29,30).

*Communication strategies that are adaptable to the patient, health care provider and local setting—*Differences in executive functioning, learning, life stage and needs among AYAs should be acknowledged and accommodated through various communication channels such as in-person meetings, social media, e-mail, handouts, websites, and mobile applications (14,19,31–37). Adolescent patients are less likely to use social media for health-related activities, preferring email rather than verbal communication methods between visits, with less concern for privacy (33). Individualized information should preferentially be provided through multiple modalities (23). Virtual methods such as telehealth visits, text messaging and/or e-mail communication should be considered for care delivery and in education initiatives.

*A defined post-transfer adult transition phase—*The existing transition literature primarily focuses on the pediatric pre-transfer phase. However, patients have different skills and capabilities at the time of transfer, resulting in different levels of readiness (38). A defined post-transfer period in a transition program allows for the full assessment of patient skills at the time of transfer and the development of a plan to ensure that any gaps are addressed.

*An evaluation of the program’s processes and outcomes and change in response to this evaluation*—A transition program should be routinely evaluated against predefined performance metrics and deficiencies should be addressed promptly with the input of relevant stakeholders (7,22,23,39–41). These metrics should be selected based on local expertise and resources. An accepted quality improvement methodology or formal clinical research process should be used to evaluate and improve the program.

***Statement 3. Transition programming should be structured according to local resources and should reflect input from local key stakeholders*** [Strong recommendation]

A multidisciplinary approach to a transition program is optimal, however, some regions may have restricted resources (42,43). In Canada, public insurance provides universal access to hospital and physician services but because health care is provincially administered, there is regional variation in access to services. Public health insurance may not reimburse patients for certain allied health services or prescription medications, and the need for additional private insurance varies by region. A transition program should account for these gaps in resource availability and public insurance. Key stakeholders to consult in the design and implementation of a transition program should include health service policy-makers and administrators, physicians (pediatric and adult gastroenterology, surgery, adolescent medicine), nurses, allied health providers (psychology, nutrition, social work, pharmacist), educators, patients and families.

***Statement 4. A pediatric to adult IBD transition of care program should implement developmentally appropriate strategies for AYAs to assess and address health-related knowledge, health-related behaviours, and transition-related skills.*** [Weak recommendation]

*Health-related knowledge—*A principal focus of a transition program should be knowledge assessment and improvement. Specific educational areas to be considered include IBD clinical information, medication and therapies (both patient-specific and general disease-related). Furthermore, practical vocational knowledge can include an understanding of the local health care system, insurance and reimbursement options, and employment and education opportunities.

*Health-related behaviours—*A transition program should ensure early assessment and support the development of behaviours thought to improve transition readiness, self-efficacy, self-activation and executive functioning. Other important program considerations include adherence, communication skills and empowerment.

*Transition-related skills—*Transition readiness, shared decision-making, and self-management (health management, emotion regulation and independence) should be assessed and developed through increased opportunities for confidential one-on-one visits with their clinicians and, when needed, connection with mental health resources and supports.

An interdisciplinary approach with serial assessments of key transition skills is optimal. Several related tools have been developed (Table 2). Better disease-specific knowledge may enhance coping in patients with IBD but does not affect adherence (44). Higher IBD-related knowledge was found to be associated with greater AYA health care satisfaction, higher AYA self-efficacy and more frequent patient-provider transition-related communication (21). High resilience was independently associated with lower disease activity and better quality of life in IBD patients (45). Although a standardized transition process can result in improvements in access to medical care, reduced acute and chronic complications, and increased rates of follow-up. There are no commonly agreed-upon quality metrics (17). The selection of assessment tools should be based on patient needs, available resources and the overall focus of the transition team.

**Table 2.** Transition-related skills or characteristics and assessment tools

Skills or characteristics	Assessment tools
IBD-specific knowledge	IBD-yourself (27)
	MyHealth Passport (46)
	IBD-KID2 (47)
Transition Readiness	Transition Readiness Assessment Questionnaire (TRAQ) (48)
	Successful Transition to Adulthood with Therapeutics (STARx) (49)
	Got-Transition (GoodToGo) (46)
	UNC TR(x)ANSITION (50)
	ON TRAC (51)
	NASPGHAN Transition Checklist (52)
Self-Efficacy and Self- Management	IBD Self-Efficacy Scale Adolescent (IBD-SES A) (39)
	HealthPROMISE (53)
	ImproveCareNow Self-management Handbook (54)
Functional Status	IBD Disk (55)
	IBD **d**isability Index (IBD-DI) (56)
Resilience	Conner-Davidson Resilience Scale (CD-RISC) (39)
Self-activation	Patient **a**ctivation **m**easure 13 Adolescent (PAM13 A) (57)
Adherence	Beliefs in Medicine (BMQ) (58)
	MMS-8 (59)
	MARS (60)

The program should be flexible and adaptable to the individual, local environment, and resources. Possible formats include individual or group events, discrete courses addressing specific skill deficiencies or generalized processes targeting overall necessary skills, or in-person versus virtual sessions. Program developers and clinicians should create reasonable expectations for transition-aged patients using the social-ecological model of AYA readiness for transition (SMART) (61–63). This type of model attempts to conceptualize the transition of care in terms of variables amenable to intervention and move beyond individual patient-related factors. Additional considerations include gender and sexuality, socioeconomic status, culture, neurocognition and intelligence, access to health care and insurance coverage.

***Statement 5. A pediatric to adult IBD transition of care program should address IBD-related adolescent issues with AYAs*** [Weak recommendation]

Adolescent-specific issues can impact overall health and IBD care in the transition process. In particular, these can include body image, self-esteem, peer influence or pressure, substance use, gender and sexuality, reproductive health and family planning, and vocational and educational planning. These should be explored confidentially and addressed through a multi-disciplinary team approach, with specialized expertise/referrals sought when needed.

A UK survey found that providers felt that they had received insufficient training related to meeting AYA’s psychosocial needs, addressing patient independence and managing parental expectations (64). A separate survey of transitioning patients identified that more than 50% did not receive education on reproductive health. Half received education on the risks of drugs and alcohol and most received no assistance related to career or vocation planning (65). Fewer than half of transition-aged patients have engaged in discussions related to mental health or sexuality (66). College students with IBD reported feeling unable to keep up with the academic workload, poor satisfaction with academic performance, more difficulty in concentrating, less efficient use of study time and lower confidence about the future, with an overall poorer adjustment to college life (67). An effective transition program should include strategies to enhance self-efficacy, and address barriers to educational performance (63). Health care providers who are unable to address these adolescent-specific areas should seek further training and/or engage additional providers.

***Statement 6. A pediatric to adult IBD transition of care program should implement strategies for parents/caregivers to support and encourage the development of independence in AYAs.*** [Strong recommendation]

Developing independence and the skills necessary for AYAs to manage their IBD is an incremental process that is usually not complete before transfer to adult care. In one study, only 45% of patients aged 19 to 21 years ordered their medication refills and only 50% picked up their medication from the pharmacy (68). While many patients can provide answers to disease-related questions (55% at age 16 to 18 years), fewer (15%) asked questions of their health care provider (68), suggesting that they may lack the ability or confidence to identify important health-related issues, or formulate and verbalize questions. Other studies noted that there was suboptimal knowledge regarding the frequency of medication refills (7,21), effects of drugs and alcohol on IBD (21), contact information for medical providers to schedule appointments or coordinate care (21,64,68), methods of self-advocacy (7,64) and disease-specific knowledge (64). A high proportion of patients fail to achieve the necessary skills before transfer, in some centres approaching 95% of patients (7). As patients age, they often develop skills to perform key tasks associated with disease management, but communication and long-term self-management skills may still be lacking (41,69).

There is limited evidence on strategies that can support parents/caregivers in encouraging the development of independence in AYAs, and there are no validated metrics for measuring the independence of AYAs. Achieving independence should be incremental, with goals appropriate for the developmental stage of the patient and parallel support for parents/caregivers. For example, organizing a staged shift of responsibility from parents to adolescent through one-on-one confidential visits with clinicians, having the adolescent develop skills related to calling in/picking up prescriptions, communicating directly with their medical team regarding their questions/concerns, rebooking appointments on their own, etc. Caregivers should be apprised of the patient’s progress toward independence and their active involvement in this process may result in further modification of the patient’s behaviour.

***Statement 7. Health care Professional training programs should integrate training in transition and create opportunities for related knowledge and skill development.*** [Strong recommendation]

Adult providers have identified challenges addressing transition-related issues such as psychosocial needs, lack of independence, and high parental expectations (64). Health care providers should have access to transition-specific training opportunities through pediatric and adult gastroenterology residency programs, pediatric and adult IBD fellowship programs, continuing medical education training, and IBD nurse training programs. Exposure to the concept of transition alone is insufficient to result in improved patient outcomes (19).

***Statement 8. Patients with pediatric-onset IBD undergoing transition of care to adult services should have access to a primary care provider***. [Strong recommendation]

Primary care providers (PCPs) (family physicians, general practitioners and nurse practitioners) are critical partners and provide continuity as AYAs move from specialized, multidisciplinary pediatric programs to adult gastroenterologists, who typically work without multidisciplinary support. Adult gastroenterologists rely on PCPs, to provide necessary preventive health care, health maintenance, coordination of care and referrals to other required specialists.

An Ontario study assessing over 2000 patients identified that nearly 350 patients were lost to adult gastroenterology follow-up (4). In British Columbia, 15% to 18% of patients did not continue with medical contact following transition, regardless of participation in a transition program (6). The primary reasons cited were difficulty in accessing the physician and the belief that ongoing physician assessments were not required. PCPs can monitor the transition process and ensure that AYAs continue their specialized IBD care with an adult gastroenterologist.

### Care Transfer Overlap

***Statement 9. A pediatric to adult IBD transition of care program should include a transition coordinator/navigator.*** [Weak recommendation]

Transition coordinators are variably defined in the literature. However, typically this is an individual whose primary task is coordinating the transfer of care between pediatric and adult care teams and supporting the development of transition-related skills. They can advocate for the patient, facilitate scheduling, and assist with financial or psychosocial issues (70,71). While this role has not been well studied in IBD, it has been examined in other childhood-onset chronic conditions (70,71). Patients with Type 1 diabetes had improved clinic attendance, improved glycemic control, fewer admissions for ketoacidosis and decreased drop-out rates from the adult clinics (72,73), while patients in transplant programs had better adherence rates (74,75). Notably, the International Association of Providers of AIDS Care (IAPAC) recommends a clear pediatric to the adult transition of care plan and the use of patient navigators to increase linkage to care and optimize health outcomes (76).

AYAs have identified care coordination as a key factor contributing to transition success. While the European Crohn’s and Colitis Organisation (ECCO) has suggested that this role is typically filled by an IBD nurse educated in pediatric IBD, social workers can also be effective (13,77,78). Roles and responsibilities may include operationalizing the program, conducting assessments, identifying and engaging local health care providers/resources, identifying eligible AYAs and tracking adherence in adult care. A single coordinator may administer both the pediatric and adult phases of transition or a separate adult care coordinator may be engaged. They may be either local or remote, shared or IBD-specific, depending on local needs, funding, geography, and the availability of professional expertise.

***Statement 10. The timing of care transfer to adult services should be flexible. Strategies should be implemented to optimize communication during the handover process between pediatric and adult IBD health care providers.*** [Strong recommendation]

Medical and psychosocial stability is ideal at the time of transfer and therefore flexibility in transfer timing is essential. In Canada, most pediatric medical centers are not permitted to care for people above their 18th birthday without special dispensation from health authorities, and pediatric gastroenterologists do not typically care for patients above this age. Therefore, adult gastroenterologists typically assume care of transition-aged patients around 18 years of age. Strategies to facilitate a successful transfer can include early referral initiation when prolonged adult wait times are anticipated, priority designation to accommodate urgent cases, and direct communication between pediatric and adult teams for complex patient-specific clinical information. While an overlap period between pediatric and adult has not improved transition outcomes, it does allow patients and caregivers the opportunity to voice concerns and make suggestions before the final transfer. In focus groups, patients noted that shared pediatric and adult clinics are helpful in facilitating information transfer and building confidence in the new adult gastroenterologist (79,80). However, the evidence is lacking for shared clinics models and associated improved health outcomes following the transition. The shared clinic model is also less feasible in the Canadian context due to a large number of community-based adult gastroenterologists and large geographic dispersion of patients after transfer to adult care. In addition, the fee-for-service reimbursement model of most adult gastroenterologists contrasts with the salaried model for pediatric gastroenterologists. Combined with long waitlists for adult providers, these factors make it less likely an adult provider will be willing or able to travel to a pediatric health center to attend a joint transition clinic.

***Statement 11. Transfer of care documents should be prepared by the pediatric team. These should include a transfer letter summarizing the individualized transition plan and a concise review of the patient’s medical history. Relevant supporting records should be included.*** [Strong recommendation]

A summary transfer letter prepared by the pediatric team should be provided in advance to the adult provider and patient, instilling confidence in transition-focused collaboration (13,71). Incomplete information transmission may lead to transfer delay and may impact therapeutic decisions and prognosis (12). In a Quebec survey, medical summaries were identified as one of the most valuable transfer tools by 84.7% of adult gastroenterologists and 62.5% of pediatric gastroenterologists (81). North American Society For Pediatric Gastroenterology, Hepatology & Nutrition (NASPGHAN) recommends that patients should bring this to their first adult appointment.

Canadian pediatric and adult IBD experts involved in transition have conducted qualitative evaluations on pertinent contents for care transfer (82). The transfer package may include a concise summary letter, copies of important consultative and investigative reports. A standard template can be used for the structure of the letter. It should include the following major headings: disease characteristics, clinical history and current status, key junctures of therapeutic history, noteworthy investigations and psychosocial history. It is important to note in the letter if any pending investigations should be conducted urgently in either adult or pediatric facility. Similarly, urgency for transfer to adult care should be specified. A thorough review of patient’s history will help pediatric care providers determine important reports to include in the package. Suggested documents include current routine laboratory, biomarkers and drug monitoring, all endoscopy and associated pathology, imaging studies and reports by key consultants. As noted previously, collaborative goal setting can lead to improved health outcomes and satisfaction. A list of topics such as care goals and expectations discussed with patient and family can also be included with the transfer summary. Such a list can be reviewed again with the receiving adult health care team after transfer.

***Statement 12. IBD transition of care networks should be developed and supported to facilitate transition and transfer planning.*** [Weak recommendation]

A transition network is the identification of transition-focused health care providers and resources across a wide geographic locale, to facilitate the sharing of knowledge and strategies to improve care and conduct research. The development of transition networks or centres can assist in the identification of local programming for patients (especially those relocating after secondary education), knowledge sharing, dissemination of best practices, facilitation of research collaboration, and the identification and recruitment of skilled individuals (especially allied health professionals who may not be readily accessible in some practice settings). Transition networks may include stakeholders involved in the planning and research of transition at the national, provincial and local organizations and should include multidisciplinary representations (Supplementary Table 1). Information related to a transition network, should be easily accessible to key stakeholders and updated regularly. While transition-specific networks have not been described in the literature, other networks of IBD health care providers (e.g., ImproveCareNow and the Canadian Child Health IBD Network) have been successful at connecting stakeholder groups (54,83).

### Adult Phase Transition

***Statement 13. The adult team engaged in a structured pediatric to adult IBD transition program should prioritize care delivery to transitioning AYAs***. [Strong recommendation]

The consensus group recognized the challenges faced by adult gastroenterologists in prioritizing care to transitioning AYAs, such as funding, time restrictions, waitlists, and reduced resources compared to HCPs in large pediatric health centres. Adult providers have expressed concern regarding limited exposure to pediatric IBD and transition-specific issues that can affect their perceptions of transition requirements and approach to care delivery (64).

The transfer of care period has been associated with an increase in emergency department utilization, outpatient care, and laboratory services (84). One study reported that during the 6 months following pediatric care, 10% of patients were hospitalized or visited the emergency department, regardless of their Transition Readiness Assessment Questionnaire (TRAQ) scores (48).

Interest in AYAs and transition care by the consulting adult gastroenterologist and access to an IBD nurse are considered important to patients (79). Early transfer referral initiation by the pediatric provider and prioritization of the transitioning AYAs by adult providers can optimize transition care. Patients have a more favourable perception of health care continuity when the initial visit with the adult provider occurs within 6 months of transfer (85). Adult gastroenterologists should be prepared for increased care requests when school is in recess, recognizing the role and participation of parent/caregiver, and more frequent calls as AYAs continue to develop their self-management skills. Specifically, the consensus group felt that the receiving adult team should be prepared to accept the expedited transfer for AYA younger than age 18 if they require urgent assessments or investigations best conducted in the adult centre, or if they are temporarily relocating to attend secondary education and to serve as either the ‘home’ team or ‘school’ team.

***Statement 14. The pediatric and adult IBD transition teams should review the processes and structure of adult health care with AYAs and parents/caregivers. The adult IBD transition team should establish expectations and goals with the AYAs and parents/guardians.*** [Strong recommendation]

Poor understanding of the differences between pediatric and adult care can impact AYAs and their family’s ability to successfully navigate the adult system. Transition programs that include modules pertaining to expectations and adult care adjustments have shown a positive effect on AYA competency and improvements in self-efficacy and satisfaction (63). Table 3 lists topics to review regarding differences in adult care.

**Table 3.** Topics to review with AYAs and families regarding adult care

By the pediatric health care team before transfer
Differences in procedural sedation How to access pediatric medical records The role of the primary care provider in IBD care The intended receiving adult gastroenterology health care team and location
By the adult health care team at intake meetings
Expectations related to IBD related care Collaborative realistic goal setting Roles of AYA in adult care Roles of parents/caregivers in adult care How, who and when to contact adult health care team How to access adult medical records Differences in procedural sedation

AYA, adolescents and young adults; IBD, inflammatory bowel disease.

### Special Considerations

***Statement 15. A pediatric to adult IBD transition of care program should accommodate groups with special needs, with the support of other specialists.*** [Weak recommendation]

Accommodations are required for patients with special needs and considerations that include AYA patients with intellectual, developmental, or other disabilities, multiple comorbidities, mental illness, and living independently. The transition team may not manage their non-IBD issues but should actively encourage PCP management and/or and referral to the appropriate services. These patients may require more frequent follow-up, additional investigations, and direct involvement of caregivers/guardians.

### Future Transition Research Topics

While formal transition programs can offer significant benefits, many aspects require further characterization and evaluation. The consensus panel recognized the absence of Level 1 evidence (controlled clinical trials) in IBD patients to support an intervention to improve the transition process. Therefore, the consensus guidelines are based primarily on observational research in IBD, and controlled clinical trials in other chronic diseases. The expert panel reviewed and prioritized future transition-related research topics (Supplementary Table 2).

## CONCLUSIONS

These 15 consensus statements on pediatric to the adult transition of care for AYAs with IBD, provide a framework for transition program implementation focusing on structure, key elements, process and evaluation. Despite the limited evidence, these statements can help guide the pediatric and adult health care teams in their efforts to successfully transition adolescents from pediatric to adult care. While many Canadian health care providers may be limited in their ability to implement all of these recommendations, we have provided statements that constitute requirements for adequate quality of care (strong recommendations), and those that may be aspirational in the context of limited resources (weak recommendations). This guidance document could be used to develop new transition programs, improve existing programs, and advocate for additional resources for caring for IBD patients. It should also be used to help further define research priorities in the transition of IBD patients. In summary, these consensus statements represent the first stage to improving the quality of care provided to AYAs with IBD transitioning from pediatric to adult care.

## SUPPLEMENTARY DATA

Supplementary data are available at Journal of the Canadian Association of Gastroenterology online.

Supplementary Table 1. Key Members of a pediatric to adult transition network

Supplementary Table 2. Suggested IBD transition-related research areas

gwab050_suppl_Supplementary_Appendix

gwab050_suppl_Supplementary_Tables
