# Supplementary material for: Canadian Consensus Statements on the Transition of Adolescents and Young Adults with Inflammatory Bowel Disease from Pediatric to Adult Care: A Collaborative Initiative Between the Canadian IBD Transition Network and Crohn’s and Colitis Canada
Source: J Can Assoc Gastroenterol. 2022 Mar 26;5(3):105–15. doi: 10.1093/jcag/gwab050 (PMC9157291; doi:10.1093/jcag/gwab050)
Supplement: gwab050_suppl_Supplementary_Appendix [file gwab050_suppl_supplementary_appendix.pdf]

Transition in Care for Inflammatory Bowel Disease

Search Strategies

2019

INDEX

4 MEDLINE Search – IBD M3 2
7. EMBASE Search – IBD E3 4
10. MEDLINE Search – IBD M7 7
11. EMBASE Search – IBD E4 9
12. MEDLINE Search – IBD M11 11
16. EMBASE Search – IBD E7 14
17. MEDLINE Search – IBD M12 minus M11 17
19. EMBASE Search – IBD E8 minus E7 23

4 MEDLINE Search – IBD M3

Database: MEDLINE (OVID)
UBC access: http://resources.library.ubc.ca/139

OVID Account: 2019IBD
Search Name: IBD M3

RefWorks: https://refworks.scholarsportal.info/Refworks/login.asp?WNCLang=false

UBC Login: ubclibref
Login account: 2019IBD
Folders: IBD M3

Date: June 8, 2019

```
Database: Ovid MEDLINE(R) and In-Process & Other Non-Indexed
Citations <1946 to June 07, 2019>
Search Strategy:
--------------------------------------------------------------
1      Inflammatory Bowel Diseases/ (20095)
2      colitis, ulcerative/ (32971)
3      crohn disease/ (37134)
4      Inflammatory Bowel Disease?.mp. (47075)
5      crohn$ disease.mp. (51581)
6      ((granulomatousl or regional) adj3 enteritis).ti,ab,kf.
(1073)
7      ileocolitis.ti,ab,kf. (415)
8      (regional adj3 (ileitis or ileitides or
enteritis)).ti,ab,kf. (1708)
9      (terminal adj3 ileitis).ti,ab,kf. (424)
10      (granulomatous adj3 enteritis).ti,ab,kf. (189)
11      (colitis adj (gravis or ulcerative or
granulomatous)).ti,ab,kf. (2790)
12      (idiopathic adj3 proctocolitis).ti,ab,kf. (36)
13      IBD.ti,ab,kf. (21760)
14      or/1-13 (101579)

15      "continuity of patient care"/ (18097)
16      transition to adult care/ (1154)
17      transitional care/ (514)
18      patient transfer/ (7772)
19      patient handoff/ (994)
20      transition$.mp. (385295)
21      "Delivery of Health Care"/ (84444)
22      transfer$.mp. (700737)
```

```
23      shared care.mp. (1211)
24      or/15-23 (1160149)

25      14 and 24 (2256)

26      adolescent/ or young adult/ (2279100)
27      Pediatrics/ (51205)
28      Adolescent Medicine/ (1499)
29      Adolescent Health/ (799)
30      Adolescent Health Services/ (5345)
31      (teen$ or youth or adolescen$).mp. (2015495)
32      young adult?.mp. (802185)
33      emerging adult?.mp. (1394)
34      (Paediatric? or pediatric?).mp. (352013)
35      (young men or young women or young male? or young
female?).ti,ab,kf. (53229)
36      (juvenile or young person? or young people or young
adulthood).ti,ab,kf. (102394)
37      or/26-36 (2704392)

38      and/14,24,37 (455)
39      comment/ or editorial/ or letter/ or news/ (1901378)
40      38 not 39 (449)
41      limit 40 to English language (422)
42      40 not 41 [Non-English] (27)

43      guideline/ or practice guideline/ (31504)
44      and/14,37,43 (38)
45      44 not 38 (33)
46      limit 45 to English language (27)
47      45 not 46 [Non-English] (6)

48      guideline?.mp. (417483)
49      14 and 37 and 48 (383)
50      49 not (38 or 44) (312)
51      comment/ or editorial/ or letter/ or news/ (1901378)
52      50 not 51 (310)
53      limit 52 to English language (284)
54      52 not 53 [Non-English] (26)
55      47 or 54 (32)
```

7. *EMBASE Search – IBD E3*

UBC access: **Database:** EMBASE (OVID)
UBC access: http://resources.library.ubc.ca/129

OVID Account: 2019IBD
Search Name: IBD E3

RefWorks: https://refworks.scholarsportal.info/Refworks/login.asp?WNCLang=false

UBC Login: ubclibref
Login account: 2019IBD
Folders: IBD 33

Search was executed on: June 8, 2019

```
Database: Embase <1980 to 2019 June 07>
Search Strategy:
--------------------------------------------------------------------------------
1     inflammatory bowel disease/ (26974)
2     crohn disease/ (79287)
3     colon crohn disease/ (2043)
4     ulcerative colitis/ (63618)
5     Inflammatory Bowel Disease?.mp. (78858)
6     crohn$ disease.mp. (89161)
7     ((granulomatousl or regional) adj3 enteritis).ti,ab,kf.
(201)
8     ileocolitis.ti,ab,kf. (474)
9     (regional adj3 (ileitis or ileitides or
enteritis)).ti,ab,kf. (238)
10     (terminal adj3 ileitis).ti,ab,kf. (395)
11     (granulomatous adj3 enteritis).ti,ab,kf. (169)
12     (colitis adj (gravis or ulcerative or
granulomatous)).ti,ab,kf. (100)
13     (idiopathic adj3 proctocolitis).ti,ab,kf. (32)
14     IBD.ti,ab,kf. (44859)
15     or/1-14 [IBD] (162523)

16     transition to adult care/ (1608)
17     transitional care/ (2141)
18     integrated health care system/ (10434)
19     transition$.mp. (473757)
20     transfer$.mp. (827764)
21     shared care.mp. (2017)
22     or/16-21 [Transition in Care] (1282099)
```

```
23      15 and 22 (5103)
24      adolescent/ (1380137)
25      young adult/ (293247)
26      adolescence/ (33992)
27      Pediatrics/ (66919)
28      Adolescent Health/ (7746)
29      child health care/ (32711)
30      (teen$ or youth or adolescen$).mp. (1497123)
31      young adult?.mp. (369018)
32      emerging adult?.mp. (1796)
33      (Paediatric? or pediatric?).mp. (540444)
34      (young men or young women or young male? or young female?).ti,ab,kf. (67092)
35      (juvenile or young person? or young people or young adulthood).ti,ab,kf. (123367)
36      or/24-35 [Adolescents] (2248364)

37      and/15,22,36 [IBD & Transition & Adolescents] (772)

38      MEDLINE.cr. (6920638)
39      37 and 38 (52)
40      37 not 39 [Remove MEDLINE records] (720)
41      remove duplicates from 40 (705)
42      editorial/ or letter/ or note/ (2298658)
43      41 not 42 (689)
44      limit 43 to English language (677)
45      43 not 44 [Other languages] (12)
46      "review"/ or "systematic review"/ (2415707)
47      meta analysis/ (163772)
48      46 or 47 [Reviews] (2484557)
49      44 and 48 [Search limited to reviews] (71)
50      44 not 49 [Remaining references] (606)

51      practice guideline/ or clinical pathway/ (385821)
52      and/15,36,51 [IBD & Adolescents & Guidelines] (342)

53      52 not 37 [Removing transition results] (313)
54      MEDLINE.cr. (6920638)
55      53 and 54 (20)
56      53 not 55 (293)
57      remove duplicates from 56 (290)
58      editorial/ or letter/ or note/ (2298658)
59      57 not 58 (275)
60      limit 59 to conference abstract status (70)
61      59 not 60 (205)
62      limit 61 to English language (188)
```

63 61 not 62 [Other languages - guidelines] (17)

10. MEDLINE Search – IBD M7

Database: MEDLINE (OVID)
UBC access: http://resources.library.ubc.ca/139

OVID Account: 2019IBD
Search Name: IBD M7

RefWorks: https://refworks.scholarsportal.info/Refworks/login.asp?WNCLang=false

UBC Login: ubclibref
Login account:
Folders: IBD M7

Date: June 19, 2019

```
Database: Ovid MEDLINE(R) and In-Process & Other Non-Indexed
Citations <1946 to June 18, 2019>
Search Strategy:
--------------------------------------------------------------
1      Rheumatology/ (6623)
2      Rheumatologists/ (159)
3      Rheumatolog$.ti,ab,kf. (27524)
4      rheumatic diseases/ (22270)
5      arthritis, juvenile/ (9963)
6      (juvenile adj4 arthritis).ti,ab,kf. (9672)
7      or/1-6 (57692)
8      transition to adult care/ (1158)
9      transitional care/ (517)
10      patient transfer/ (7778)
11      transition$.mp. (389071)
12      shared care.ti,ab,kf. (1212)
13      (transfer$ adj7 (youth or adolescen$ or teen$)).ti,ab,kf.
(411)
14      (transfer$ adj7 (adult$ or care or health)).ti,ab,kf.
(12078)
15      (transfer$ adj7 (pediatric or paediatric or child$ or
young or juvenile?)).ti,ab,kf. (4455)
16      or/8-15 (410607)

17      7 and 16 (372)
18      limit 17 to English language (329)
19      17 not 18 [Non-English] (43)
20      comment/ or editorial/ or letter/ or news/ (1904978)
21      18 and 20 (17)
22      18 not 21 (312)
```

```
23      "systematic review"/ or "review"/ (2501099)
24      22 and 23 (59)
25      22 not 24 (253)
```

11. EMBASE Search – IBD E4

UBC access: **Database:** EMBASE (OVID)
UBC access: http://resources.library.ubc.ca/129

OVID Account: 2019IBD
Search Name: IBD E4

RefWorks: https://refworks.scholarsportal.info/Refworks/login.asp?WNCLang=false

UBC Login: ubclibref
Login account:
Folders: IBD E4

Search was executed on: June 19, 2019

```
Database: Embase <1974 to 2019 June 18>
Search Strategy:
--------------------------------------------------------------------------------
1      Rheumatology/ (63173)
2      Rheumatologist/ (3324)
3      Rheumatolog$.ti,ab,kw. (71914)
4      rheumatic disease/ (40617)
5      juvenile rheumatoid arthritis/ (18981)
6      (juvenile adj4 arthritis).ti,ab,kw. (16025)
7      or/1-6 (142113)
8      transition to adult care/ (1621)
9      transitional care/ (2164)
10      integrated health care system/ (10458)
11      transition$.mp. (483076)
12      shared care.ti,ab,kw. (2020)
13      (transfer$ adj7 (youth or adolescen$ or teen$)).ti,ab,kw.
(709)
14      (transfer$ adj7 (adult$ or care or health)).ti,ab,kw.
(20290)
15      (transfer$ adj7 (pediatric or paediatric or child$ or
young or juvenile?)).ti,ab,kw. (6649)
16      or/8-15 (518344)

17      7 and 16 (1770)

18      adolescent/ (1433762)
19      young adult/ (294756)
20      adolescence/ (36182)
21      Pediatrics/ (72008)
```

22 Adolescent Health/ (7785)
23 child health care/ (35243)
24 (teen$ or youth or adolescen$).mp. (1557287)
25 young adult?.mp. (373030)
26 emerging adult?.mp. (1808)
27 (Paediatric? or pediatric?).mp. (555721)
28 (young men or young women or young male? or young female?).ti,ab,kw. (70309)
29 (juvenile or young person? or young people or young adulthood).ti,ab,kw. (132084)
30 or/18-29 [Adolescents] (2335392)

31 7 and 16 and 30 (700)

32 MEDLINE.cr. (7550032)
33 31 and 32 (29)
34 31 not 33 (671)

35 limit 34 to conference abstract status (431)
36 34 not 35 (240)
37 limit 36 to English language (207)
38 36 not 37 [Non-Eng] (33)
39 "review"/ or "systematic review"/ (2483937)
40 37 and 39 [Reviews] (44)
41 37 not 40 (163)
42 remove duplicates from 35 (428)

12. *MEDLINE Search – IBD M11*

Database: MEDLINE (OVID)
UBC access: http://resources.library.ubc.ca/139

OVID Account: 2019IBD
Search Name: IBD M11

RefWorks: https://refworks.scholarsportal.info/Refworks/login.asp?WNCLang=false

UBC Login: ubclibref
Login account: 2019IBD
Folders: IBD M11

Date: July 11, 2019

```
Database: Ovid MEDLINE(R) and In-Process & Other Non-Indexed
Citations <1946 to July 10, 2019>
Search Strategy:
--------------------------------------------------------------
1     transition to adult care/ (1175)
2     transitional care/ (533)
3     patient transfer/ (7813)
4     transition$.mp. (391047)
5     shared care.ti,ab,kf. (1214)
6     (transfer$ adj7 (youth or adolescen$ or teen$)).ti,ab,kf.
(412)
7     (transfer$ adj7 (adult$ or care or health)).ti,ab,kf.
(12125)
8     (transfer$ adj7 (pediatric or paediatric or child$ or
young or juvenile?)).ti,ab,kf. (4474)
9     or/1-8 (412663)

10    *Transition to Adult Care/st [Standards] (86)
11    *Transition to Adult Care/og [Organization &
Administration] (244)
12    or/10-11 (329)

13    Gastroenterology/ (9893)
14    Gastroenterologists/ (233)
15    Gastrointestinal Diseases/th [Therapy] (3581)
16    or/13-15 (13376)

17    9 and 16 (98)

18    Physician-Patient Relations/ (70136)
```

19 Adolescent Health Services/og [Organization & Administration] (1757)
20 Interprofessional Relations/ (49837)
21 Patient Education as Topic/ (82292)
22 Continuity of Patient Care/ (18181)
23 og.fs. [Organization & Administration] (458835)
24 Attitude of Health Personnel/ (116112)
25 st.fs. [standards] (683858)
26 practice guideline/ (24820)
27 position paper?.mp. (3351)
28 Patient-Centered Care/ (17727)
29 mt.fs. [methods] (3595482)
30 "Health Services Needs and Demand"/ (51371)
31 Delivery of Health Care/ (85019)
32 or/18-31 (4655826)

33 adolescent/ or young adult/ (2289750)
34 Pediatrics/ (51352)
35 Adolescent Medicine/ (1501)
36 Adolescent Health/ (811)
37 Adolescent Health Services/ (5376)
38 (teen$ or youth or adolescen$).mp. (2023312)
39 young adult?.mp. (810472)
40 emerging adult?.mp. (1422)
41 (Paediatric? or pediatric?).mp. (354410)
42 (young men or young women or young male? or young female?).ti,ab,kf. (53498)
43 (juvenile or young person? or young people or young adulthood).ti,ab,kf. (103014)
44 or/33-43 (2718185)

45 guideline/ or practice guideline/ (31635)
46 *Patient Care Planning/ (13583)
47 guideline?.mp. (420864)
48 Recommendation?.mp. (228830)
49 (Strategy or Strategies).mp. (937459)
50 st.fs. [standards] (683858)
51 program$.ti,ab,kf. (809664)
52 (Tool or tools).ti,ab,kf. (633910)
53 (protocol or protocols).ti,ab,kf. (394252)
54 (method or methods).ti,ab,kf. (5594706)
55 px.fs. [Psychology] (996906)
56 time factors/ (1155574)
57 (model or models).ti,ab,kf. (2453204)
58 (approach or approaches).ti,ab,kf. (1571495)
59 skill?.ti,ab,kf. (161365)

60 (experience? or barrier? or challeng$ or success$ or skill? or concern? or perspective? or insight?).ti,ab,kf. (3548644)
61 Quality indicator?.ti,ab,kf. (7470)
62 (goal or goals).ti,ab,kf. (306182)
63 Interdisciplinary Communication/ (16265)
64 partnership.ti,ab,kf. (21309)
65 co-ordination.ti,ab,kf. (3438)
66 og.fs. [Organization & Administration] (458835)
67 Practice Guidelines as Topic/ (110666)
68 position paper?.mp. (3351)
69 Age Factors/ (435735)
70 patient care management/ (3661)
71 critical pathways/ (6323)
72 disease management/ (33096)
73 patient care team/ (62889)
74 "delivery of health care"/ (85019)
75 checklist?.ti,ab,kf. (33841)
76 or/45-75 (12880027)

77 *Cystic Fibrosis/ (28254)
78 *Diabetes Mellitus, Type 1/ (59543)
79 *Asthma/ (99466)
80 *Cerebral Palsy/ (15923)
81 *Heart Defects, Congenital/ (39444)
82 or/77-81 [Diseases combined] (242051)

83 9 and 81 and 44 and (32 or 76) [Heart defects] (204)
84 9 and 80 and 44 and (32 or 76) [Cerebral Palsy] (127)
85 9 and 79 and 44 and (32 or 76) [Asthma] (124)
86 9 and 77 and 44 and (32 or 76) [Cystic Fibrosis] (141)
87 9 and 78 and 44 and (32 or 76) [Diabetes] (316)

88 9 and **82** and 44 and (32 or 76) [Various Diseases] (904)
89 9 and 16 and 44 and (32 or 76) [Gastroenterology] (45)
90 **12** and 44 [Transition to adult care] (317)
91 88 or 89 or 90 [Searches Combined] (1207)

92 remove duplicates from 91 (1207)
93 comment/ or editorial/ or letter/ or news/ (1911577)
94 92 not 93 (1170)
95 limit 94 to English language (1077)
96 "systematic review"/ or "review"/ (2510644)
97 95 and 96 (202)
98 95 not 97 [remaining records] (875)
99 94 not 95 [Other languages] (93)
100 92 not 94 (37)

16. EMBASE Search – IBD E7

UBC access: **Database:** EMBASE (OVID)
UBC access: http://resources.library.ubc.ca/129

OVID Account: 2019IBD
Search Name: IBD E7

RefWorks: https://refworks.scholarsportal.info/Refworks/login.asp?WNCLang=false

UBC Login: ubclibref
Login account: 2019IBD
Folders: IBD E7

Search was executed on: July 11, 2019

```
Database: Embase <1974 to 2019 July 10>
Search Strategy:
--------------------------------------------------------------
1      gastroenterology/ (49069)
2      gastroenterologist/ (6079)
3      gastrointestinal disease/dm, th [Disease Management,
Therapy] (2194)
4      or/1-3 (55819)

5      *transition to adult care/ (868)

6      *cystic fibrosis/ (44555)
7      *insulin dependent diabetes mellitus/ (67841)
8      *asthma/ or *allergic asthma/ or *asthmatic state/ or
*moderate persistent asthma/ or *severe persistent asthma/
(150200)
9      *cerebral palsy/ (20195)
10      exp *congenital heart disease/ or congenital heart
malformation/ (101919)
11      or/6-10 [Other Diseases] (383842)

12      insulin dependent diabetes mellitus/dm, th [Disease
Management, Therapy] (6393)
13      or/6,8-10,12 [Other Diseases] (322581)

14      transition to adult care/ (1639)
15      transitional care/ (2211)
16      integrated health care system/ (10520)
17      transition$.mp. (486209)
```

18 shared care.ti,ab,kw. (2024)
19 (transfer$ adj7 (youth or adolescen$ or teen$)).ti,ab,kw. (714)
20 (transfer$ adj7 (adult$ or care or health)).ti,ab,kw. (20453)
21 (transfer$ adj7 (pediatric or paediatric or child$ or young or juvenile?)).ti,ab,kw. (6685)
22 or/14-21 (521701)

23 4 and 22 [Gastroenterology] (932)
24 **5** and **11** [Transition in care] (134) **Search #1**
25 13 and 22 [Other diseases] (3075)

26 adolescent/ (1439366)
27 young adult/ (298097)
28 adolescence/ (36405)
29 Pediatrics/ (72391)
30 Adolescent Health/ (7826)
31 child health care/ (35333)
32 (teen$ or youth or adolescen$).mp. (1563598)
33 young adult?.mp. (376646)
34 emerging adult?.mp. (1851)
35 (Paediatric? or pediatric?).mp. (559134)
36 (young men or young women or young male? or young female?).ti,ab,kw. (70618)
37 (juvenile or young person? or young people or young adulthood).ti,ab,kw. (132795)
38 or/26-37 [Adolescents] (2347088)

39 4 and 22 and 38 [Gastroenterology] (252)
40 13 and 22 and 38 [Other diseases] (1415)

41 practice guideline/ (383846)
42 clinical pathway/ or consensus development/ or good clinical practice/ (40704)
43 guideline?.mp. (691816)
44 patient care planning/ (27665)
45 Recommendation?.mp. (328308)
46 program$.ti,ab,kw. (1066762)
47 (Tool or tools).ti,ab,kw. (872908)
48 (protocol or protocols).ti,ab,kw. (592998)
49 (method or methods).ti,ab,kw. (8394237)
50 (model or models).ti,ab,kw. (3184682)
51 (approach or approaches).ti,ab,kw. (1969294)
52 skill?.ti,ab,kw. (221063)

53 (experience? or barrier? or challeng$ or success$ or skill? or concern? or perspective? or insight?).ti,ab,kw. (4665015)
54 Quality indicator?.ti,ab,kw. (12241)
55 interdisciplinary communication/ (11587)
56 partnership.ti,ab,kw. (30052)
57 co-ordination.ti,ab,kw. (4482)
58 position paper?.mp. (4195)
59 *patient care/ (64161)
60 checklist?.ti,ab,kw. (47575)
61 doctor patient relation/ (112743)
62 health personnel attitude/ (76270)
63 or/41-62 (14645761)

64 4 and 22 and 38 and 63 [Gastroenterology] (226) **Search #2**
65 13 and 22 and 38 [Other diseases] (1415) **Search #3**

66 or/24,64-65 [All 3 Searches] (1701)

67 MEDLINE.cr. (7569883)
68 66 and 67 (187)
69 66 not 68 (1514)
70 limit 69 to conference abstract status (771)
71 69 not 70 (743)
72 limit 71 to English language (684)
73 71 not 72 [Non-English] (59)
74 editorial/ or letter/ or note/ (2362186)
75 72 and 74 (27)
76 72 not 75 (657)
77 "systematic review"/ or "review"/ (2495001)
78 76 and 77 (129)
79 76 not 78 [Remaining English] (528)

17. *MEDLINE Search – IBD M12 minus M11*

Database: MEDLINE (OVID)
UBC access: http://resources.library.ubc.ca/139

OVID Account: 2019IBD
Search Name: IBD M12

RefWorks: https://refworks.scholarsportal.info/Refworks/login.asp?WNCLang=false

UBC Login: ubclibref
Login account: 2019IBD
Folders: IBD M12

Date: July 11, 2019

```
Database: Ovid MEDLINE(R) and In-Process & Other Non-Indexed
Citations <1946 to July 10, 2019>
Search Strategy:
--------------------------------------------------------------
```

IBD M12 Search

```
1     hiv/ or hiv-1/ or hiv-2/ (95256)
2     exp Multiple Sclerosis/ (55570)
3     exp Epilepsy/ (107270)
4     or/1-3 (257521)

5     transition to adult care/ (1175)
6     4 and 5 (53)  Search #1

7     *hiv/ or *hiv-1/ or *hiv-2/ (73072)
8     exp *Multiple Sclerosis/ (47758)
9     exp *Epilepsy/ (88261)
10     or/7-9 (208887)

11     transition to adult care/ (1175)
12     transitional care/ (533)
13     patient transfer/ (7813)
14     transition$.mp. (391047)
15     shared care.ti,ab,kf. (1214)
16     (transfer$ adj7 (youth or adolescen$ or teen$)).ti,ab,kf.
(412)
17     (transfer$ adj7 (adult$ or care or health)).ti,ab,kf.
(12125)
18     (transfer$ adj7 (pediatric or paediatric or child$ or
young or juvenile?)).ti,ab,kf. (4474)
19     or/11-18 (412663)
```

20 Physician-Patient Relations/ (70136)
21 Adolescent Health Services/og [Organization & Administration] (1757)
22 Interprofessional Relations/ (49837)
23 Patient Education as Topic/ (82292)
24 Continuity of Patient Care/ (18181)
25 og.fs. [Organization & Administration] (458835)
26 Attitude of Health Personnel/ (116112)
27 st.fs. [standards] (683858)
28 practice guideline/ (24820)
29 position paper?.mp. (3351)
30 Patient-Centered Care/ (17727)
31 mt.fs. [methods] (3595482)
32 "Health Services Needs and Demand"/ (51371)
33 Delivery of Health Care/ (85019)
34 or/20-33 (4655826)

35 adolescent/ or young adult/ (2289750)
36 Pediatrics/ (51352)
37 Adolescent Medicine/ (1501)
38 Adolescent Health/ (811)
39 Adolescent Health Services/ (5376)
40 (teen$ or youth or adolescen$).mp. (2023312)
41 young adult?.mp. (810472)
42 emerging adult?.mp. (1422)
43 (Paediatric? or pediatric?).mp. (354410)
44 (young men or young women or young male? or young female?).ti,ab,kf. (53498)
45 (juvenile or young person? or young people or young adulthood).ti,ab,kf. (103014)
46 or/35-45 (2718185)

47 guideline/ or practice guideline/ (31635)
48 *Patient Care Planning/ (13583)
49 guideline?.mp. (420864)
50 Recommendation?.mp. (228830)
51 (Strategy or Strategies).mp. (937459)
52 st.fs. [standards] (683858)
53 program$.ti,ab,kf. (809664)
54 (Tool or tools).ti,ab,kf. (633910)
55 (protocol or protocols).ti,ab,kf. (394252)
56 (method or methods).ti,ab,kf. (5594706)
57 px.fs. [Psychology] (996906)
58 time factors/ (1155574)
59 (model or models).ti,ab,kf. (2453204)
60 (approach or approaches).ti,ab,kf. (1571495)

61 skill?.ti,ab,kf. (161365)
62 (experience? or barrier? or challeng$ or success$ or skill? or concern? or perspective? or insight?).ti,ab,kf. (3548644)
63 Quality indicator?.ti,ab,kf. (7470)
64 (goal or goals).ti,ab,kf. (306182)
65 Interdisciplinary Communication/ (16265)
66 partnership.ti,ab,kf. (21309)
67 co-ordination.ti,ab,kf. (3438)
68 og.fs. [Organization & Administration] (458835)
69 Practice Guidelines as Topic/ (110666)
70 position paper?.mp. (3351)
71 Age Factors/ (435735)
72 patient care management/ (3661)
73 critical pathways/ (6323)
74 disease management/ (33096)
75 patient care team/ (62889)
76 "delivery of health care"/ (85019)
77 checklist?.ti,ab,kf. (33841)
78 or/47-77 (12880027)

79 10 and 19 and 46 (350)
80 10 and 19 and 46 and (34 or 78) (279) **Search #2**

81 6 or 80 [Searches 1 and 2] (297)

82 remove duplicates from 81 (297)
83 limit 82 to humans (**291**) **Search Results M12**

IBD M11 Search
84 transition to adult care/ (1175)
85 transitional care/ (533)
86 patient transfer/ (7813)
87 transition$.mp. (391047)
88 shared care.ti,ab,kf. (1214)
89 (transfer$ adj7 (youth or adolescen$ or teen$)).ti,ab,kf. (412)
90 (transfer$ adj7 (adult$ or care or health)).ti,ab,kf. (12125)
91 (transfer$ adj7 (pediatric or paediatric or child$ or young or juvenile?)).ti,ab,kf. (4474)
92 or/84-91 (412663)
93 *Transition to Adult Care/st [Standards] (86)
94 *Transition to Adult Care/og [Organization & Administration] (244)
95 or/93-94 (329)
96 Gastroenterology/ (9893)

97 Gastroenterologists/ (233)
98 Gastrointestinal Diseases/th [Therapy] (3581)
99 or/96-98 (13376)
100 92 and 99 (98)
101 Physician-Patient Relations/ (70136)
102 Adolescent Health Services/og [Organization & Administration] (1757)
103 Interprofessional Relations/ (49837)
104 Patient Education as Topic/ (82292)
105 Continuity of Patient Care/ (18181)
106 og.fs. [Organization & Administration] (458835)
107 Attitude of Health Personnel/ (116112)
108 st.fs. [standards] (683858)
109 practice guideline/ (24820)
110 position paper?.mp. (3351)
111 Patient-Centered Care/ (17727)
112 mt.fs. [methods] (3595482)
113 "Health Services Needs and Demand"/ (51371)
114 Delivery of Health Care/ (85019)
115 or/101-114 (4655826)
116 adolescent/ or young adult/ (2289750)
117 Pediatrics/ (51352)
118 Adolescent Medicine/ (1501)
119 Adolescent Health/ (811)
120 Adolescent Health Services/ (5376)
121 (teen$ or youth or adolescen$).mp. (2023312)
122 young adult?.mp. (810472)
123 emerging adult?.mp. (1422)
124 (Paediatric? or pediatric?).mp. (354410)
125 (young men or young women or young male? or young female?).ti,ab,kf. (53498)
126 (juvenile or young person? or young people or young adulthood).ti,ab,kf. (103014)
127 or/116-126 (2718185)
128 guideline/ or practice guideline/ (31635)
129 *Patient Care Planning/ (13583)
130 guideline?.mp. (420864)
131 Recommendation?.mp. (228830)
132 (Strategy or Strategies).mp. (937459)
133 st.fs. [standards] (683858)
134 program$.ti,ab,kf. (809664)
135 (Tool or tools).ti,ab,kf. (633910)
136 (protocol or protocols).ti,ab,kf. (394252)
137 (method or methods).ti,ab,kf. (5594706)
138 px.fs. [Psychology] (996906)
139 time factors/ (1155574)
140 (model or models).ti,ab,kf. (2453204)

141 (approach or approaches).ti,ab,kf. (1571495)
142 skill?.ti,ab,kf. (161365)
143 (experience? or barrier? or challeng$ or success$ or skill? or concern? or perspective? or insight?).ti,ab,kf. (3548644)
144 Quality indicator?.ti,ab,kf. (7470)
145 (goal or goals).ti,ab,kf. (306182)
146 Interdisciplinary Communication/ (16265)
147 partnership.ti,ab,kf. (21309)
148 co-ordination.ti,ab,kf. (3438)
149 og.fs. [Organization & Administration] (458835)
150 Practice Guidelines as Topic/ (110666)
151 position paper?.mp. (3351)
152 Age Factors/ (435735)
153 patient care management/ (3661)
154 critical pathways/ (6323)
155 disease management/ (33096)
156 patient care team/ (62889)
157 "delivery of health care"/ (85019)
158 checklist?.ti,ab,kf. (33841)
159 or/128-158 (12880027)
160 *Cystic Fibrosis/ (28254)
161 *Diabetes Mellitus, Type 1/ (59543)
162 *Asthma/ (99466)
163 *Cerebral Palsy/ (15923)
164 *Heart Defects, Congenital/ (39444)
165 or/160-164 (242051)
166 92 and 164 and 127 and (115 or 159) [Heart defects] (204)
167 92 and 163 and 127 and (115 or 159) [Cerebral Palsy] (127)
168 92 and 162 and 127 and (115 or 159) [Asthma] (124)
169 92 and 160 and 127 and (115 or 159) [Cystic Fibrosis] (141)
170 92 and 161 and 127 and (115 or 159) [Diabetes] (316)
171 92 and 165 and 127 and (115 or 159) (904)
172 92 and 99 and 127 and (115 or 159) [Gastroenterology] (45)
173 95 and 127 [Transition to adult care] (317)
174 171 or 172 or 173 (1207)
175 remove duplicates from 174 (1207)
176 comment/ or editorial/ or letter/ or news/ (1911577)
177 175 not 176 (1170)
178 limit 177 to English language (1077)
179 "systematic review"/ or "review"/ (2510644)
180 178 and 179 (202)
181 178 not 180 [remaining records] (875)

182 177 not 178 [Other languages] (93)

183 83 not 174 (278) **IBD M2 minus M11**
184 limit 183 to English language (255)
185 183 not 184 [Non-English] (23)

19. EMBASE Search – IBD E8 minus E7

Database: EMBASE (OVID)
UBC access: http://resources.library.ubc.ca/129

OVID Account: 2019IBD
Search Name: IBD E8 minus E7

RefWorks: https://refworks.scholarsportal.info/Refworks/login.asp?WNCLang=false

UBC Login: ubclibref
Login account: 2019IBD
Folders: IBD E8

Search was executed on: July 11, 2019

```
Database: Embase <1974 to 2019 July 11>
Search Strategy:
--------------------------------------------------------------------------------
1     exp epilepsy/dm, th [Disease Management, Therapy] (8540)
2     multiple sclerosis/dm, th [Disease Management, Therapy]
(5570)
3     exp Human immunodeficiency virus/ (187385)
4     1 or 2 or 3 (201384)

5     exp Epilepsy/ (220163)
6     multiple sclerosis/ (118281)
7     exp Human immunodeficiency virus/ (187385)
8     or/5-7 (521711)

9     transition to adult care/ (1639)
10     8 and 9 (83)

11     transition to adult care/ (1639)
12     transitional care/ (2214)
13     integrated health care system/ (10522)
14     transition$.mp. (486385)
15     shared care.ti,ab,kw. (2027)
16     (transfer$ adj7 (youth or adolescen$ or teen$)).ti,ab,kw.
(714)
17     (transfer$ adj7 (adult$ or care or health)).ti,ab,kw.
(20464)
18     (transfer$ adj7 (pediatric or paediatric or child$ or
young or juvenile?)).ti,ab,kw. (6686)
```

19 or/11-18 (521894)

20 adolescent/ (1439574)
21 young adult/ (298266)
22 adolescence/ (36429)
23 Pediatrics/ (72410)
24 Adolescent Health/ (7826)
25 child health care/ (35333)
26 (teen$ or youth or adolescen$).mp. (1563862)
27 young adult?.mp. (376826)
28 emerging adult?.mp. (1854)
29 (Paediatric? or pediatric?).mp. (559301)
30 (young men or young women or young male? or young female?).ti,ab,kw. (70639)
31 (juvenile or young person? or young people or young adulthood).ti,ab,kw. (132829)
32 or/20-31 [Adolescents] (2347639)

33 practice guideline/ (384004)
34 clinical pathway/ or consensus development/ or good clinical practice/ (40704)
35 guideline?.mp. (692056)
36 patient care planning/ (27665)
37 Recommendation?.mp. (328465)
38 program$.ti,ab,kw. (1067182)
39 (Tool or tools).ti,ab,kw. (873345)
40 (protocol or protocols).ti,ab,kw. (593287)
41 (method or methods).ti,ab,kw. (8398511)
42 (model or models).ti,ab,kw. (3186218)
43 (approach or approaches).ti,ab,kw. (1970237)
44 skill?.ti,ab,kw. (221152)
45 (experience? or barrier? or challeng$ or success$ or skill? or concern? or perspective? or insight?).ti,ab,kw. (4667052)
46 Quality indicator?.ti,ab,kw. (12246)
47 interdisciplinary communication/ (11588)
48 partnership.ti,ab,kw. (30068)
49 co-ordination.ti,ab,kw. (4484)
50 position paper?.mp. (4196)
51 *patient care/ (64169)
52 checklist?.ti,ab,kw. (47592)
53 doctor patient relation/ (112743)
54 health personnel attitude/ (76271)
55 or/33-54 (14651888)

56 4 and 19 and 32 (372)
57 4 and 19 and 32 and 55 (329)

58 limit 57 to conference abstract status (201)
59 remove duplicates from 58 [Search #2] (200)

60 57 not 58 (128)
61 medline.cr. (7582193)
62 60 and 61 (24)
63 60 not 62 (104)
64 remove duplicates from 63 [Search #2a] (103)

65 limit 10 to conference abstract status (16)
66 10 not 65 (68)
67 66 and 61 (9)
68 66 not 67 [Search #1] (59)
69 59 or 68 [Search #1 and #2] (**259**)
70 64 not 69 [Search #2a] (**92**)

71 gastroenterology/ (49074)
72 gastroenterologist/ (6082)
73 gastrointestinal disease/dm, th [Disease Management, Therapy] (2198)
74 or/71-73 (55827)
75 *transition to adult care/ (870)
76 *cystic fibrosis/ (44604)
77 *insulin dependent diabetes mellitus/ (67996)
78 *asthma/ or *allergic asthma/ or *asthmatic state/ or *moderate persistent asthma/ or *severe persistent asthma/ (150375)
79 *cerebral palsy/ (20214)
80 exp *congenital heart disease/ or congenital heart malformation/ (102084)
81 or/76-80 (383917)
82 insulin dependent diabetes mellitus/dm, th [Disease Management, Therapy] (6407)
83 or/76,78-80,82 (322635)
84 transition to adult care/ (1645)
85 transitional care/ (2230)
86 integrated health care system/ (10546)
87 transition$.mp. (487564)
88 shared care.ti,ab,kw. (2029)
89 (transfer$ adj7 (youth or adolescen$ or teen$)).ti,ab,kw. (716)
90 (transfer$ adj7 (adult$ or care or health)).ti,ab,kw. (20518)
91 (transfer$ adj7 (pediatric or paediatric or child$ or young or juvenile?)).ti,ab,kw. (6701)
92 or/84-91 (521894)

93 74 and 92 [Gastroenterology] (933)
94 75 and 81 [Transition in care] (134)
95 83 and 92 [Other diseases] (3075)
96 adolescent/ (1442891)
97 young adult/ (300762)
98 adolescence/ (36436)
99 Pediatrics/ (72544)
100 Adolescent Health/ (7842)
101 child health care/ (35382)
102 (teen$ or youth or adolescen$).mp. (1567464)
103 young adult?.mp. (379504)
104 emerging adult?.mp. (1858)
105 (Paediatric? or pediatric?).mp. (560938)
106 (young men or young women or young male? or young female?).ti,ab,kw. (70776)
107 (juvenile or young person? or young people or young adulthood).ti,ab,kw. (133077)
108 or/96-107 [Adolescents] (2347639)
109 74 and 92 and 108 [Gastroenterology] (252)
110 83 and 92 and 108 [Other diseases] (1415)
111 practice guideline/ (385188)
112 clinical pathway/ or consensus development/ or good clinical practice/ (40771)
113 guideline?.mp. (694264)
114 patient care planning/ (27693)
115 Recommendation?.mp. (329377)
116 program$.ti,ab,kw. (1069728)
117 (Tool or tools).ti,ab,kw. (875778)
118 (protocol or protocols).ti,ab,kw. (595111)
119 (method or methods).ti,ab,kw. (8422119)
120 (model or models).ti,ab,kw. (3194615)
121 (approach or approaches).ti,ab,kw. (1975306)
122 skill?.ti,ab,kw. (221663)
123 (experience? or barrier? or challeng$ or success$ or skill? or concern? or perspective? or insight?).ti,ab,kw. (4678870)
124 Quality indicator?.ti,ab,kw. (12279)
125 interdisciplinary communication/ (11608)
126 partnership.ti,ab,kw. (30137)
127 co-ordination.ti,ab,kw. (4490)
128 position paper?.mp. (4216)
129 *patient care/ (64345)
130 checklist?.ti,ab,kw. (47720)
131 doctor patient relation/ (112754)
132 health personnel attitude/ (76476)
133 or/111-132 (14651888)
134 74 and 92 and 108 and 133 [Gastroenterology] (226)

135 83 and 92 and 108 [Other diseases] (1415)
136 or/94,134-135 (1701)
137 MEDLINE.cr. (7573270)
138 136 and 137 (187)
139 136 not 138 (1514)
140 limit 139 to conference abstract status (771)
141 139 not 140 (743)
142 limit 141 to English language (684)
143 141 not 142 [Non-English] (59)
144 editorial/ or letter/ or note/ (2362728)
145 142 and 144 (27)
146 142 not 145 (657)
147 "systematic review"/ or "review"/ (2495562)
148 146 and 147 (129)
149 146 not 148 (528)

150 69 not 136 (253)
151 limit 150 to English language (245) **– Search #1-2**
152 150 not 151 [Non-English] (7)
153 70 not 136 (90)
154 limit 153 to English language (88) **– Search #2a**
155 from 154 keep 12,25,39,78 (4)
156 from 155 keep 1,3 (2)

Note

Only line 151 was exported to Excel and a text file for EndNote
