# Supplementary material for: Canadian Consensus Statements on the Transition of Adolescents and Young Adults with Inflammatory Bowel Disease from Pediatric to Adult Care: A Collaborative Initiative Between the Canadian IBD Transition Network and Crohn’s and Colitis Canada
Source: J Can Assoc Gastroenterol. 2022 Mar 26;5(3):105–15. doi: 10.1093/jcag/gwab050 (PMC9157291; doi:10.1093/jcag/gwab050)
Supplement: gwab050_suppl_Supplementary_Tables [file gwab050_suppl_supplementary_tables.docx]

**Supplementary Table 1: Key Members of a pediatric to adult transition network**

| Medical | |
| --- | --- |
| Primary | Pediatric gastroenterology |
|  | Adult gastroenterology |
|  | IBD Nursing |
| Associated | Adolescent medicine |
|  | Colorectal surgery |
|  | Dermatology |
|  | Rheumatology |
|  | Ophthalmology |
|  | Psychiatry |
| Allied Health | |
|  | Psychology |
|  | Dietician/nutrition |
|  | Social work |

**Supplementary Table 2: Suggested IBD transition-related research areas**

| Transition assessment tools | Patient independence, transition care needs, progression though transition stages, completion of transition, psychosocial risk factors related to transition, knowledge on adolescent issues, IBD and transition (eg. sexual function, family planning, cannabis) |
| --- | --- |
| Transition model of care | Objective comparison between different models of care, barriers to the uptake and implementation of a transition program, roles of stakeholders (including primary care providers, transition navigators), strategies for late adolescent-onset IBD |
| Transition quality indicators | Transition success, quality of life, patient satisfaction, medication adherence, transfer of care wait times, loss to adult IBD follow-up, emergent health resource utilization |
| Operationalizing a transition clinic | Requirements of a transfer letter, methods for medical data transfer, methods to discourage unnecessary health resource utilization |
| Transition network | The impact of a transition network, the role of transition network, necessary stakeholders |
